# cAMP-Inhibits Cytoplasmic Phospholipase A_2_ and Protects Neurons against Amyloid-β-Induced Synapse Damage

**DOI:** 10.3390/biology4030591

**Published:** 2015-09-16

**Authors:** Clive Bate, Alun Williams

**Affiliations:** 1 Department of Pathology and Pathogen Biology, Royal Veterinary College, Hawkshead Lane, North Mymms, Herts AL9 7TA, UK; 2 Department of Veterinary Medicine, University of Cambridge, Madingley Road, Cambridge CB3 OES, UK; E-Mail: aw510@cam.ac.uk

**Keywords:** Alzheimer’s disease, amyloid, cAMP, phosphodiesterase, phospholipase A_2_, prostaglandin E_2_, synapses

## Abstract

A key event in Alzheimer’s disease (AD) is the production of amyloid-β (Aβ) peptides and the loss of synapses. In cultured neurons Aβ triggered synapse damage as measured by the loss of synaptic proteins. α-synuclein (αSN), aggregates of which accumulate in Parkinson’s disease, also caused synapse damage. Synapse damage was associated with activation of cytoplasmic phospholipase A_2_ (cPLA_2_), an enzyme that regulates synapse function and structure, and the production of prostaglandin (PG) E_2_. In synaptosomes PGE_2_ increased concentrations of cyclic adenosine monophosphate (cAMP) which suppressed the activation of cPLA_2_ demonstrating an inhibitory feedback system. Thus, Aβ/αSN-induced activated cPLA_2_ produces PGE_2_ which increases cAMP which in turn suppresses cPLA_2_ and, hence, its own production. Neurons pre-treated with pentoxifylline and caffeine (broad spectrum phosphodiesterase (PDE) inhibitors) or the PDE4 specific inhibitor rolipram significantly increased the Aβ/αSN-induced increase in cAMP and consequently protected neurons against synapse damage. The addition of cAMP analogues also inhibited cPLA_2_ and protected neurons against synapse damage. These results suggest that drugs that inhibit Aβ-induced activation of cPLA_2_ and cross the blood–brain barrier may reduce synapse damage in AD.

## 1. Introduction

The amyloid hypothesis maintains that the key event in the pathogenesis of Alzheimer’s disease (AD) is the proteolytic cleavage of the amyloid precursor protein to form neurotoxic amyloid-β (Aβ) peptides [1]. The accumulation of Aβ leads to the disruption of neuronal processes, abnormal phosphorylation of tau and synapse dysfunction. The progressive dementia associated with AD is thought to be due to synaptic failure [2] and a reduction in synaptic proteins correlates strongly with cognitive decline [3,4].

Some of the events that lead to neurodegeneration in AD can be examined by incubating cultured neurons with Aβ peptides. Soluble Aβ oligomers are recognized as the primary mediators of synapse damage and dementia has been recognized [5,6] and in these studies soluble Aβ oligomers derived from the brain of an Alzheimer’s patient [7] were used. The effects of Aβ oligomers on neurons were examined by measuring the amounts of synaptic proteins, as a marker of synapse density [8]. The Aβ oligomers were active at picomolar concentrations, similar to the concentrations of Aβ found within the brain [9]. Synapse damage is also a feature of other neurodegenerative diseases including Parkinson’s disease (PD) and dementia with Lewy bodies [10]. The pathology of these diseases is associated with the formation of oligomeric α-synuclein (αSN) [11,12] and preparations of αSN oligomers trigger synapse damage in cultured neurons [13].

In the current study, we show that phosphodiesterase (PDE) inhibitors protect cultured neurons against Aβ and αSN-induced synapse damage. The protective effect of PDE inhibitors was related to its effects upon the concentrations of cyclic adenosine monophosphate (cAMP) within synapses. Thus, PDE inhibitors enhanced the Aβ/αSN-induced increase of cAMP in synapses. High concentrations of cAMP suppressed the Aβ/αSN-induced activation of cytoplasmic phospholipase A_2_ (cPLA_2_), a key enzyme in synapse function and structure [14]. In addition, we demonstrate the presence of an inhibitory feedback system whereby prostaglandin (PG)E_2_, a product of cPLA_2_ activation, increased concentrations of cAMP within synapses. The cAMP inhibited the activation of cPLA_2_ and hence its own production.

## 2. Experimental Section

### 2.1. Primary Cortical Neuronal Cultures

Neurons were prepared from the cerebral cortices of mouse embryos (day 15.5) as described [14]. Neurons were plated at 2 × 10^5^ cells/well in 48 well plates (coated with poly-L-lysine) in Ham’s F12 containing 5% foetal calf serum for 2 h. Cultures were shaken (600 r.p.m for 5 min) and non-adherent cells removed by 3 washes in PBS. Neurons were then grown in neurobasal medium containing B27 components supplemented with 5 nM brain derived nerve growth factor for 10 days. Immunohistochemistry showed that the cells were greater than 90% neurofilament positive. All experiments were performed in accordance with European regulations (European Community Council Directive, 1986, 56/609/EEC) and approved by the Royal Veterinary College ethical committee.

Neurons were pre-treated with test compounds for 1 h before the addition of peptides or a PLA_2_ isolated from the venom of the Mozambique spitting cobra (Sigma, Poole, UK) for 24 h. They were then homogenized in an extraction buffer containing 150 mM NaCl, 10 mM Tris-HCl pH 7.4, 10 mM EDTA, 0.5% Nonidet P-40, 0.5% sodium deoxycholate, 0.2% sodium dodecyl sulphate (SDS) and mixed protease inhibitors (4-(2-Aminoethyl) benzenesulfonyl fluoride hydrochloride, Aprotinin, Leupeptin, Bestatin, Pepstatin A and E-46) (Sigma) and a phosphatase inhibitor cocktail including PP1, PP2A, microcystin LR, cantharidin and p-bromotetramisole (Sigma) at 10^6^ cells/mL. Nuclei and cell debris was removed by centrifugation (1000× *g* for 5 min).

### 2.2. Isolation of Synaptosomes

Synaptosomes were prepared from 10^6^ neurons homogenized at 4 °C in 1 mL of SED solution (0.32 M sucrose, 50 mM Tris-HCl pH 7.2, 1 mM EDTA, and 1 mM dithiothreitol). The supernatant was transferred to a 4-step gradient of 3%, 7%, 15%, and 23% Percoll in SED solution and centrifuged at 16,000× *g* for 30 min at 4 °C. The synaptosomes were collected from the interface of the 15% and 23% Percoll steps, washed (16,000× *g* for 30 min at 4 °C) and suspended in neurobasal medium containing B27 components. Freshly prepared synaptosomes were pre-treated ±drugs and incubated with peptides for 1 h. Treated synaptosomes were homogenized in extraction buffer (as above). The amounts of cAMP within synaptosomes were determined using a kit (Enzo Life Sciences, Farmingdale, NY, USA).

### 2.3. Activated cPLA_2_ ELISA

The activation of cPLA_2_ is accompanied by phosphorylation of the 505 serine residue creating a unique epitope that can be recognized by specific antisera. Maxisorb immunoplates (Nunc, Roskilde, Denmark) were coated with 0.5 µg/mL of the mouse anti-cPLA_2_ monoclonal antibody (mAb) (clone CH-7 (Upstate)) and then blocked with 10% milk powder. Samples were added for 1 h and bound activated cPLA_2_ was detected using rabbit polyclonal anti-phospho-cPLA_2_ (Cell Signaling Technology, Danvers, MA, USA) followed by biotinylated anti-rabbit IgG (Sigma), extravidin-alkaline phosphatase and 1 mg/mL 4-nitrophenyl phosphate. Samples were expressed as “units activated cPLA_2_” where 1 units was the amount of activated cPLA_2_ in control synaptosomes.

### 2.4. Synaptophysin ELISA

The amounts of synaptophysin in neurons were measured by ELISA as described [8]. Maxisorb immunoplates were coated with an anti-synaptophysin mouse mAb MAB368 (Millipore, Billerica, MA, USA) and blocked with 5% milk powder. Samples were added for 1 h and bound synaptophysin was detected using rabbit polyclonal anti-synaptophysin antibodies (Abcam, Cambridge, UK) followed by a biotinylated anti-rabbit IgG, extravidin-alkaline phosphatase and 1 mg/mL 4-nitrophenol phosphate. Absorbance was measured on a microplate reader at 405 nm. Samples were expressed as “units synaptophysin” where 100 units was defined as the amount of synaptophysin in 10^6^ control neurons.

### 2.5. Western Blotting

Samples were mixed with Laemmli buffer containing β-mercaptoethanol, heated to 95 °C for 5 min and proteins were separated by electrophoresis on 15% polyacrylamide gels (PAGE). Proteins were transferred onto a Hybond-P PVDF membrane by semi-dry blotting. Membranes were blocked using 10% milk powder; synaptophysin was detected with MAB368 (Abcam), vesicle-associated membrane protein (VAMP)-1 with mAb 4H302 (Abcam), synapsin-1 with a rabbit polyclonal antibody (515200, Invitrogen, Waltham, MA, USA) and caveolin with rabbit polyclonal antibodies (Upstate, Damstadt, Germany). These were visualized using a combination of biotinylated anti-mouse/rat/rabbit IgG (Sigma), extravidin-peroxidase and enhanced chemiluminescence.

### 2.6. Peptides

Recombinant human αSN and βSN were obtained from Sigma. Stock solutions of peptides were thawed on the day of use and mixed in culture medium. Mixtures were subjected to vigorous shaking (disruptor genie, full power for 10 min) before they were added to neurons.

### 2.7. Preparation of Aβ-Containing Medium

The temporal lobe from a 78 year old female with a clinical, and pathologically-confirmed, diagnosis of Alzheimer’s disease, was supplied by Asterand, an international supplier of human tissue. Soluble extracts were prepared using methodology as described [15]. Briefly, brain tissue was cut into pieces of approximately 100 mg and added to 2 mL tubes containing lysing matrix D beads (Q-Bio, Cambridge, UK). Neurobasal medium containing B27 components was added so that there was the equivalent of 100 mg brain tissue/mL. The tubes were shaken for 10 min (Disruptor genie, Scientific Instruments, Oxford, UK) for 3 times and tubes centrifuged at 16,000× *g* for 10 min to remove cell debris. Soluble material was prepared by passage through a 50 kDa filter (Sartorius, Damstadt, Germany). The amounts of Aβ in each soluble extract were measured by ELISA (see below) and the supernatant stored at −80 °C.

### 2.8. Immunodepletions

Brain extracts were incubated with mAb 4G8 (reactive with amino acids 17–24 of Aβ) and incubated at 4 °C on rollers for 1 h. Protein G microbeads (Sigma) were added (10 µL/mL) for 30 min and protein G-antibody complexes were removed by centrifugation (16,000× *g* for 10 min). Mock-depletions involved incubating brain extracts with an isotype control followed by protein G microbeads under the same conditions as above. Depleted media was filtered before further use.

### 2.9. Aβ_42_ ELISA

Maxisorb immunoplates were coated with mAb 4G8 (epitope 17–24) (Covance, Princeton, NJ, USA). Plates were blocked with 5% milk powder in PBS-tween and samples were applied. The detection antibody was an Aβ_42_ selective rabbit mAb BA3-9 (Covance) followed by biotinylated anti-rabbit IgG (Sigma), extravidin-alkaline phosphatase and 1 mg/mL 4-nitrophenol phosphate. Absorbance was read in a spectrophotometer at 405 nm. Results were calculated by comparison to synthetic Aβ_42_ (Bachem, Bubendorf, Switzerland).

### 2.10. Aβ_40_ ELISA

Maxisorb immunoplates were coated with mAb 4G8 (epitope 17–24) (Covance, Maidenhead, UK). Plates were blocked with 5% milk powder and samples were applied. The detection antibody was an Aβ_40_ selective rabbit polyclonal PC-149 (Merck, Damstadt, Germany) followed by biotinylated anti-rabbit IgG, extravidin alkaline phosphatase and 1 mg/mL 4-nitrophenol phosphate, Absorbance was read in a spectrophotometer at 405 nm. Results were calculated by comparison to serial dilutions of synthetic Aβ_40_ (Bachem, Bubendorf, Switzerland).

### 2.11. Drugs

Arachidonyl trifluoromethyl ketone (AACOCF_3_), aspirin, caffeine, pentoxifylline, AH13205, BWA868C, dibutyryl cAMP, 8-bromo-cAMP, ibuprofen, methyl arachidonyl fluorophosphonate (MAFP) and prostaglandins D_2_, E_2_, I_2_ and J_2_ were obtained from Sigma. Stock solutions were dissolved in ethanol or di-methyl sulphoxide and diluted in neurobasal medium to obtain final working concentrations. Control medium consisted of equivalent dilutions of ethanol or di-methyl sulphoxide in neurobasal medium.

### 2.12. Statistical Methods

Differences between treatment groups were assessed using Student’s, paired *t*-tests.

## 3. Results and Discussion

### 3.1. Aβ Triggered Synapse Damage

**Figure 1:**
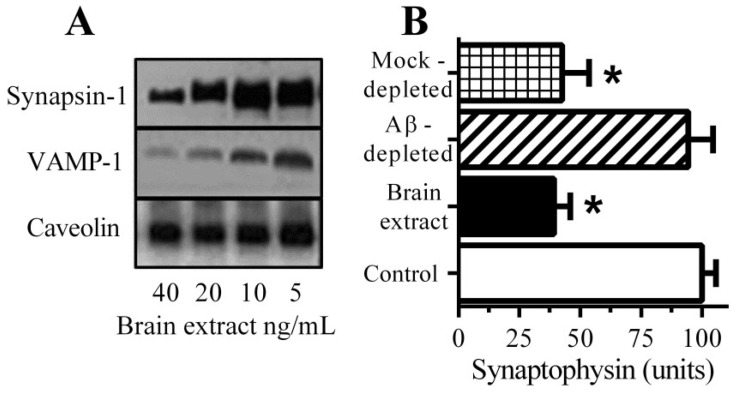
Aβ causes synapse damage in cultured neurons: (**A**) Immunoblots showing the amounts of synapsin-1, VAMP-1 and caveolin in neurons incubated with brain extract as shown; (**B**) The amounts of synaptophysin in neurons incubated with control medium (□), brain extract (■), Aβ-depleted brain extract (striped bar) or mock-depleted brain extract (hatched bar). Values are means ± SD from triplicate experiments performed 2 times (*n* = 6). * = synaptophysin significantly less than in control neurons.

Immunoblots of neuronal extracts showed that the addition of brain extract causes the loss of synapsin-1 and vesicle-associated membrane protein (VAMP)-1 (Figure 1A). They did not affect the amounts of caveolin from these neurons suggesting that synapse degeneration occurred in the absence of any significant neuronal death. Incubation with a brain extract containing 1 nM Aβ_42_ caused a significant reduction in the synaptophysin content of neurons (Figure 1B). Brain extract depleted of Aβ had reduced concentrations of both Aβ_40_ (4.9 nM ± 0.3 to 0.3 nM ± 0.3 nM) and Aβ_42_ (1.2 nM ± 0.16 to 0.11 nM ± 0.1 nM) and did not cause a significant loss of synaptophysin from neurons. These observations show that Aβ is a major toxin in brain extract preparations.

### 3.2. Pentoxifylline Protects Neurons against Aβ-Induced Synapse Damage

**Figure 2:**
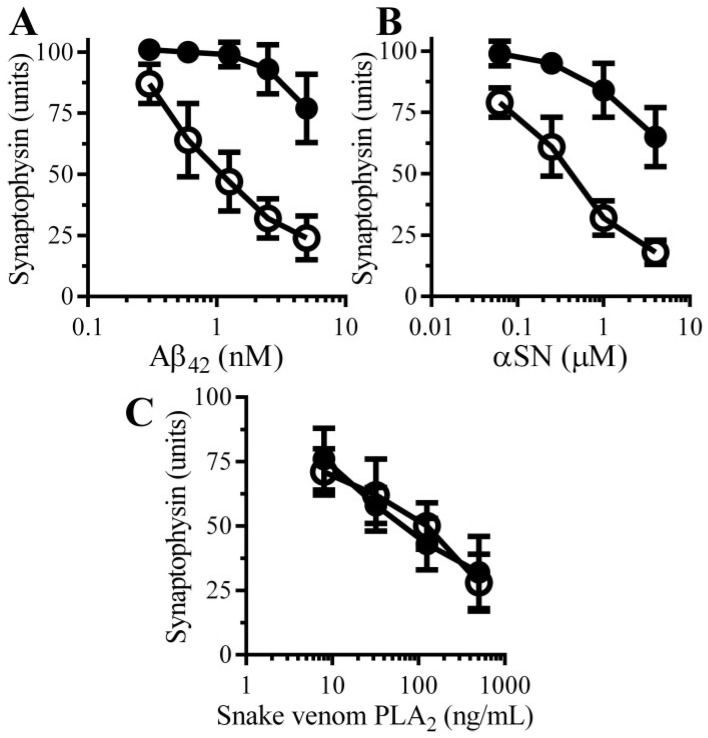
Pentoxifylline protects neurons against Aβ and αSN-induced synapse damage: The amounts of synaptophysin in neurons pre-treated with control medium (○) or 100 µM pentoxifylline (●) and incubated with Aβ_42_ (**A**), αSN (**B**) or snake venom PLA_2_ (**C**) as shown. Values are means ± SD from triplicate experiments performed 3 times (*n* = 9).

The effects of pentoxifylline, a broad spectrum PDE inhibitor, on Aβ-induced synapse damage in cultured neurons was studied. Firstly, the addition of 100 µM pentoxifylline alone did not affect synapses in cultured neurons (101 units synaptophysin ± 5 compared to 100 units synaptophysin ± 5, *n* = 9, *p* = 0.5). Pre-treatment with 100 µM pentoxifylline protected neurons against the Aβ-induced loss of synaptophysin (Figure 2A). The loss of synapses is a pathological feature that is also seen in cases of PD that show dementia [10] in which small oligomers of αSN accumulate at synapses [11]. The addition of αSN triggered the loss of synaptophysin from neurons in a tissue culture model of the synapse damage that occurs in Parkinson’s disease and dementia with Lewy bodies [13]. Pre-treatment with 100 μM pentoxifylline protected neurons against the αSN-induced loss of synaptophysin (Figure 2B). However, the loss of synaptophysin from neurons incubated with a PLA_2_ isolated from the venom of the Mozambique spitting cobra) was not significantly altered by pre-treatment with pentoxifylline (Figure 2C).

### 3.3. Caffeine and Rolipram Protect Neurons against Aβ and αSN-Induced Synapse Damage

**Figure 3:**
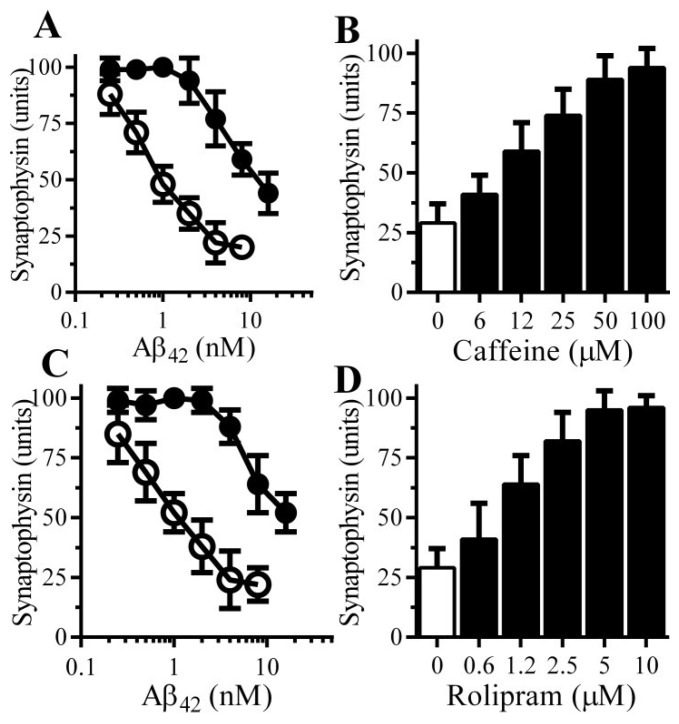
Caffeine and Rolipram protect neurons against Aβ-induced synapse damage: (**A**) The amounts of synaptophysin in neurons pre-treated with control medium (○) or 100 µM caffeine (●) and incubated with Aβ_42_ as shown. Values are means ± SD from triplicate experiments performed 4 times (*n* = 12); (**B**) The amounts of synaptophysin in neurons pre-treated with control medium (□) or caffeine as shown (■) and incubated with 2 nM Aβ_42_. Values are means ± SD from triplicate experiments performed 3 times (*n* = 9); (**C**) The amounts of synaptophysin in neurons pre-treated with control medium (○) or 10 μM rolipram (●) and incubated with Aβ_42_ as shown. Values are means ± SD from triplicate experiments performed 4 times (*n* = 12); (**D**) The amounts of synaptophysin in neurons pre-treated with control medium (□) or rolipram as shown (■) and incubated with 2 nM Aβ_42_. Values are means ± SD from triplicate experiments performed 3 times (*n* = 9).

Other PDE inhibitors were tested, pre-treatment with 100 μM caffeine protected neurons against the Aβ-induced loss of synaptophysin (Figure 3A); the effects of caffeine were dose-dependent (Figure 3B). Rolipram, which selectively inhibits PDE4 [16], also protected neurons against the Aβ-induced loss of synaptophysin (Figure 3C). The effects of rolipram were also dose-dependent (Figure 3D).

### 3.4. PDE Inhibitors Do Not Alter the Accumulation of Aβ_42_ in Synapses

The possibility that PDE inhibitors prevented synapse damage by reducing the accumulation of Aβ_42_ within synapses was explored. Neurons treated with PDE inhibitors, incubated with 10 nM Aβ_42_ for 1 h and synaptosomes were isolated from these neurons. There was no significant difference in the concentrations of Aβ_42_ found in synaptosomes isolated from neurons treated with control medium and 100 μM pentoxifylline (2.74 nM Aβ_42_ ± 0.5 compared with 2.87 ± 0.29, *n* = 9, *p* = 0.53) or 10 μM rolipram (2.74 nM Aβ_42_ ± 0.5 compared with 2.63 ± 0.45, *n* = 9, *p* = 0.45).

### 3.5. Aβ and αSN Increase the Concentrations of cAMP in Synaptosomes

**Figure 4:**
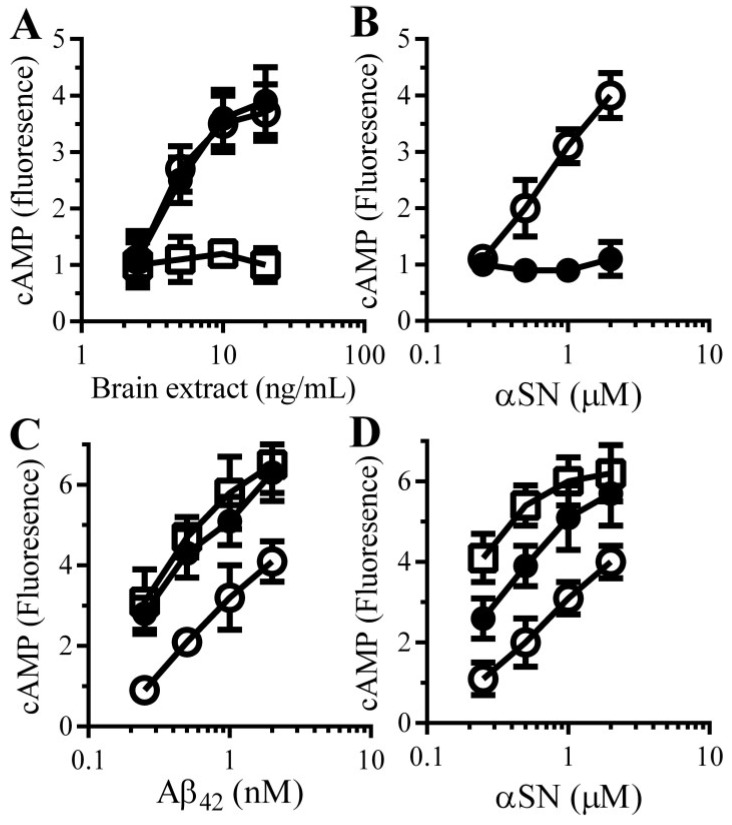
PDE inhibitors enhance the Aβ and αSN-induced increase in synaptic cAMP concentrations in synaptosomes: (**A**) The amounts of cAMP in synaptosomes incubated with brain extract (○), Aβ-depleted brain extract (□) or mock-depleted brain extract (●). Values are means ± SD, from triplicate experiments performed 3 times (*n* = 9); (**B**) The amounts of cAMP in synaptosomes incubated with αSN (○) or βSN (●). Values are means ± SD, from triplicate experiments performed 3 times (*n* = 9). The amounts of cAMP in synaptosomes pre-treated with control medium (○), 100 μM pentoxifylline (●) or 10 μM rolipram (□) and incubated with Aβ_42_ (**C**) or αSN (**D**) as shown. Values are means ± SD, from triplicate experiments performed 3 times (*n* = 9).

These results implied that the protective effect of PDE inhibitors were mediated via its inhibition of the PDEs and increased cAMP concentrations. To test this hypothesis we first examined the effects of Aβ and αSN on cAMP concentrations in synaptosomes. The addition of brain extract (Figure 4A) or αSN (Figure 4B) increased the concentrations of cAMP within synaptosomes in a dose-dependent manner, whereas the addition of control preparations (Aβ-depleted brain extract or βSN) had no significant effect. Next, the effect of pentoxifylline and rolipram on the concentration of cAMP in synaptosomes was measured. cAMP concentrations in control synaptosomes were not significantly affected by 100 µM pentoxifylline (0.97 units cAMP ± 0.12 compared with 1 ± 0.92, *n* = 9, *p* = 0.68) or 10 μM rolipram (1.05 units cAMP ± 0.16 compared with 1 ± 0.92, *n* = 9, *p* = 0.51). Pre-treatment with either 100 µM pentoxifylline or 10 μM rolipram increased cAMP concentrations in synaptosomes incubated with Aβ (Figure 4C) or αSN (Figure 4D).

### 3.6. cAMP Analogues Protect Neurons against Aβ and αSN-Induced Synapse Damage

**Figure 5:**
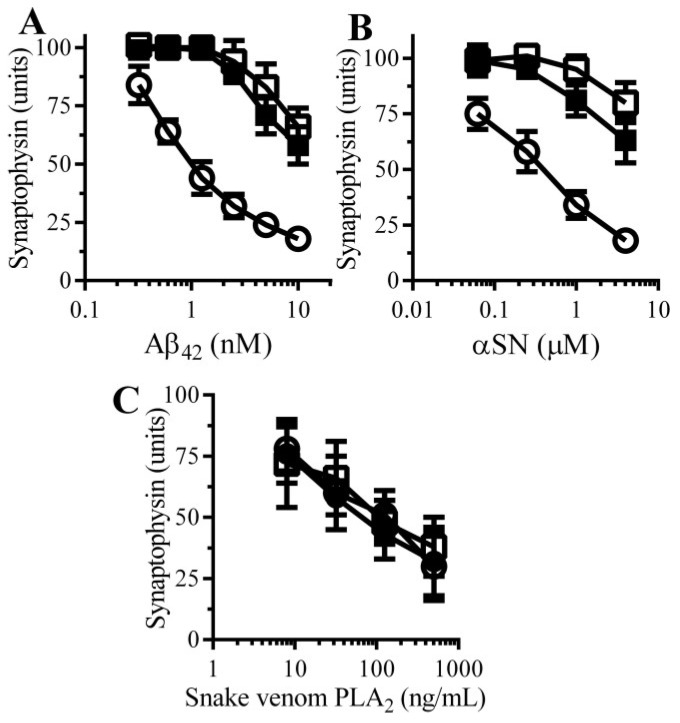
cAMP protects neurons against Aβ and αSN-induced synapse damage: The amounts of synaptophysin in neurons pre-treated with control medium (○), 100 µM dibutyryl cAMP (□) or 100 µM 8-bromo-cAMP (■) and incubated with Aβ_42_ (**A**); αSN (**B**) or snake venom PLA_2_ (**C**) as shown. Values are means ± SD from triplicate experiments performed 3 times (*n* = 9).

To complement the PDE inhibitor studies; the effects of two cAMP analogues (dibutyryl cAMP and 8-bromo-cAMP) on neurons were studied. The amounts of synaptophysin in neuronal cultures was not significantly affected by incubation with either 100 μM dibutyryl cAMP (98 units synaptophysin ± 7 compared with 100 units ± 6, *n* = 9, *p* = 0.4) or 100 μM 8-bromo-cAMP (97 units synaptophysin ± 8 compared with 100 units ± 6, *n* = 9, *p* = 0.35) indicating that these drugs alone did not damage synapses. Pre-treatment with 100 μM dibutyryl cAMP or 100 μM 8-bromo-cAMP protected neurons against the loss of synaptophysin induced by either Aβ (Figure 5A) or αSN (Figure 5B), but had no effect against snake-venom PLA_2_-induced synapse degeneration (Figure 5C).

### 3.7. PDE Inhibitors Reduce the Aβ and αSN-Induced Activation of cPLA_2_ in Synapses

**Figure 6:**
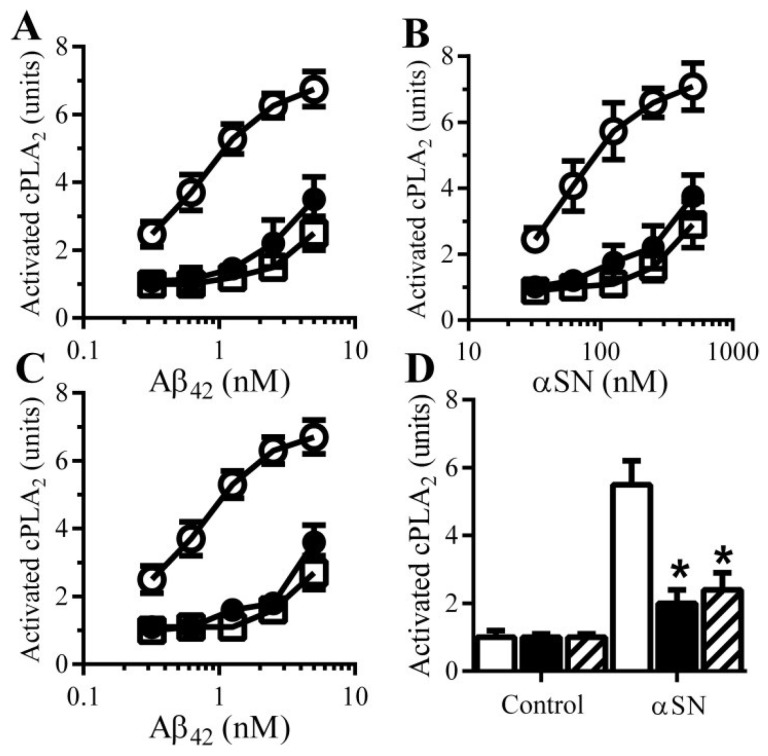
cAMP reduces Aβ and αSN-induced activation of cPLA_2_: The amounts of activated cPLA_2_ in synaptosomes pre-treated with control medium (○), 100 μM pentoxifylline (●) or 10 μM rolipram (□) and incubated with Aβ_42_ (**A**) or αSN (**B**) as shown. Values are means ± SD from triplicate experiments performed 3 times (*n* = 9); (**C**) The amounts of activated cPLA_2_ in synaptosomes pre-treated with control medium (○), 100 µM dibutyryl cAMP (□) or 100 µM 8-bromo-cAMP (●) and incubated with Aβ_42_ as shown. Values are means ± SD from triplicate experiments performed 3 times (*n* = 9); (**D**) The amounts of activated cPLA_2_ in synaptosomes pre-treated with control medium (□), 100 µM dibutyryl cAMP (■) or 100 µM 8-bromo-cAMP (striped bars) and incubated with 200 nM αSN. Values are means ± SD from triplicate experiments performed 3 times (*n* = 9).

There is increasing evidence to implicate Aβ-induced activation of specific cell signaling pathways in synapse damage. For example, Aβ_42_ activates cPLA_2_ [17,18] and inhibition of cPLA_2_ protects cultured neurons against Aβ-induced synapse damage [14] and ameliorated cognitive decline in a mouse model of AD [19]. For these reasons the effects of PDE inhibitors and cAMP analogues on cPLA_2_ in synaptosomes were examined. The amounts of activated cPLA_2_ in synaptosomes was not significantly altered by treatment with 100 µM pentoxifylline (1.06 units cAMP ± 0.15 compared with 1 ± 0.14, *n* = 9, *p* = 0.52) or 10 μM rolipram (1.07 units cAMP ± 0.12 compared with 1 ± 0.92, *n* = 9, *p* = 0.44). The addition of Aβ or αSN increased the amounts of activated cPLA_2_ within synaptosomes [14]. Pre-treatment of synaptosomes with 100 μM pentoxifylline or 10 μM rolipram significantly reduced the activation of cPLA_2_ by Aβ (Figure 6A) and αSN (Figure 6B). Similarly, pre-treatment with 100 μM dibutyryl cAMP or 8-bromo-cAMP reduced the activation of cPLA_2_ by Aβ (Figure 6C) and by αSN (Figure 6D).

### 3.8. PGE_2_-Mediates Aβ-Induced Increase in Camp

**Figure 7:**
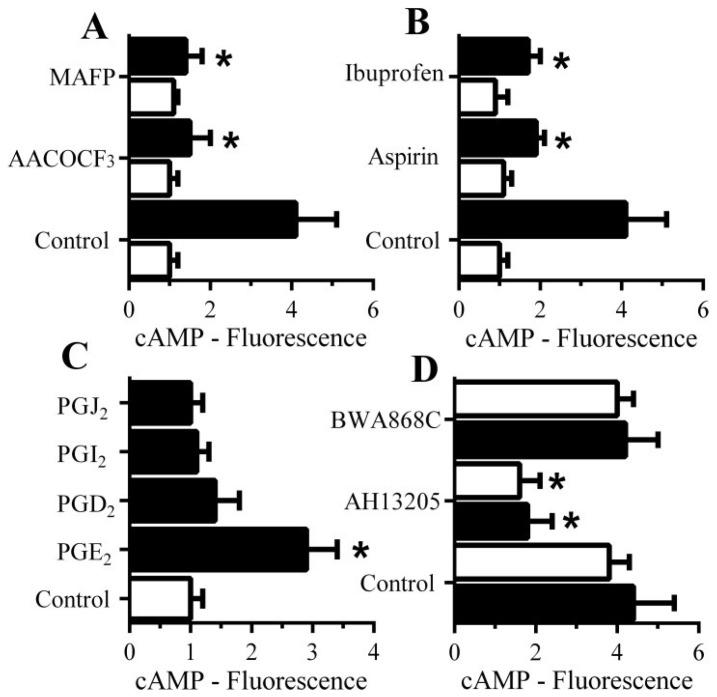
The Aβ-induced increase in synaptic cAMP is caused by PGE_2_: (**A**) The amounts of cAMP in synaptosomes pre-treated with control medium, 1 μM AACOCF_3_ or 1 μM MAFP and incubated with control medium (□) or 1 nM Aβ_42_ (■). Values are means ± SD, from triplicate experiments performed twice (*n* = 6). * = cAMP significantly lower than synaptosomes incubated with Aβ; (**B**) The amounts of cAMP in synaptosomes pre-treated with control medium, 1 μM aspirin or 1 μM Ibuprofen and incubated with control medium (□) or 1 nM Aβ_42_ (■). Values are means ± SD, from triplicate experiments performed twice (*n* = 6). * = cAMP lower than synaptosomes incubated with Aβ; (**C**) The amounts of cAMP in synaptosomes incubated with control medium (□) or 1 nM prostaglandins as shown (■). Values are means ± SD, from triplicate experiments performed twice (*n* = 6). * = cAMP significantly higher than control synaptosomes; (**D**) The amounts of cAMP in synaptosomes pre-treated with control medium, 100 nM AH13205 (a prostanoid E receptor antagonist) or 100 nM BWA868C (a prostanoid D receptor antagonist) and incubated with 1 nM Aβ_42_ (■) or 100 nM αSN (□). Values are means ± SD, from triplicate experiments performed twice (*n* = 6).

Since cAMP inhibited the activation of cPLA_2_ and reduced synapse degeneration, the molecular mechanisms leading to cAMP generation within synaptosomes were examined in detail. The Aβ-induced increase in cAMP was significantly reduced by pre-treatment with cPLA_2_ inhibitors (1 μM AACOCF_3_ or 1 μM MAFP) (Figure 7A) and by the cyclo-oxygenase inhibitors aspirin and ibuprofen (Figure 7B) suggesting that a PG is involved in the Aβ-induced increase in cAMP. The addition of 1 nM PGE_2_, but not other PGs, significantly increased cAMP concentrations in synaptosomes (Figure 7C). Since PGs exert their effects through specific receptors, synaptosomes were pre-treated with selective antagonists of prostanoid receptors. Pre-treatment of synaptosomes with the prostanoid E receptor antagonist AH13205 significantly reduced Aβ and αSN-induced increase in cAMP (Figure 7D). Pre-treatment of synaptosomes with the prostanoid D receptor antagonist BWA868C did not affect the Aβ or αSN-induced increase in cAMP concentrations.

### 3.9. PGE_2_ Inhibits the Activation of cPLA_2_ in Synapses

**Figure 8:**
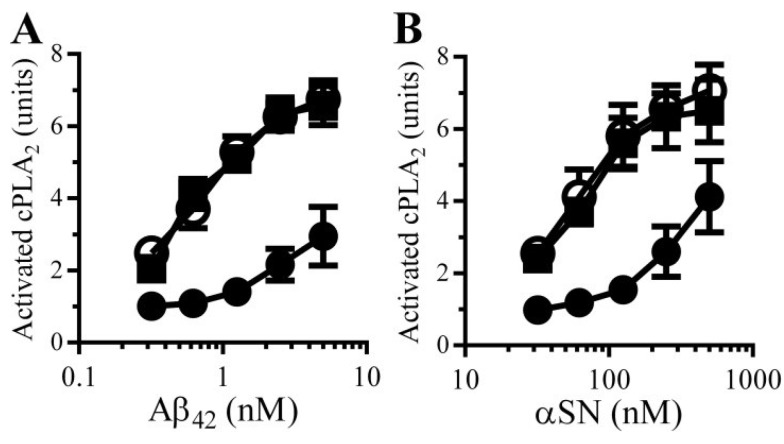
PGE_2_ reduces Aβ and αSN-induced activation of synaptic cPLA_2_: The amounts of activated cPLA_2_ in synaptosomes pre-treated for with control medium (○), 1 nM PGE_2_ (●) or 1 nM PGD_2_ (■) and incubated with Aβ_42_ (**A**) or αSN (**B**) as shown. Values are means ± SD from triplicate experiments performed 3 times (*n* = 9).

**Figure 9:**
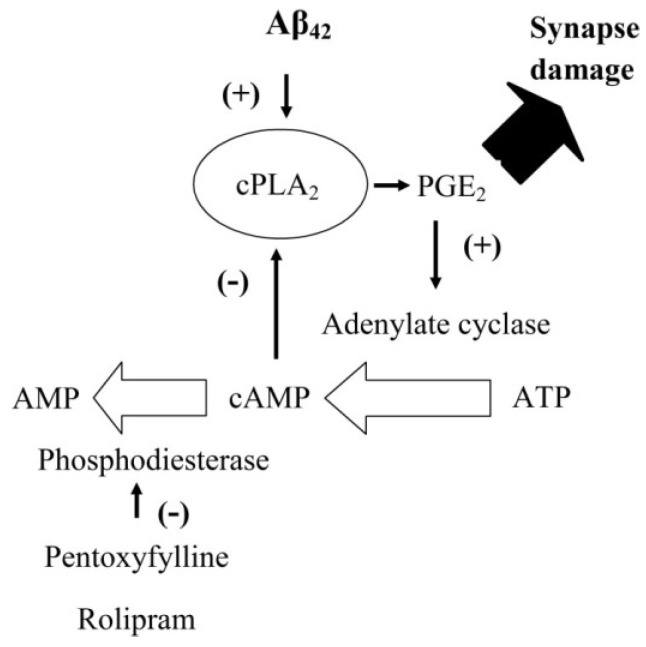
Feedback inhibition of cPLA_2_ by cAMP: Cartoon showing the proposed inhibitory feedback system that regulates the activation of cPLA_2_ in synapses. The presence of Aβ activates cPLA_2_ leading to the production of PGE_2_ high concentrations of which cause synapse damage. However, PGE_2_ also increases concentrations of cAMP which in turn inhibits the activation of cPLA_2_ completing an inhibitory feedback system. In control synapses cAMP is degraded by PDEs. Inhibition of PDEs by either pentoxifylline or rolipram results in higher concentrations of cAMP within synapses. Higher cAMP concentrations have a stronger inhibitory feedback signal and reduce the Aβ-induced activation of cPLA_2_.

PGE_2_ is involved in synapse function and regulates synaptic plasticity [20]. The observation that PGE_2_ increased the concentrations of cAMP in synaptosomes and that cAMP suppresses Aβ and αSN-induced synapse damage suggests the presence of an inhibitory feedback system within synapses. We hypothesised that the Aβ/αSN-induced activation of cPLA_2_ results in the production of PGE_2_ which increases cAMP and inhibits cPLA_2_ activation (Figure 9). This hypothesis was tested by treating synaptosomes with either 1 nM PGE_2_ or 1 nM PGD_2_ prior to incubation with Aβ or αSN. Pre-treatment of synaptosomes with PGE_2_, but not PGD_2_, significantly reduced both Aβ (Figure 8A) and αSN (Figure 8B)-induced activation of cPLA_2_.

## 4. Discussion

Neurons were incubated with disease-related peptides to model the synapse damage that is a prominent feature of AD and Parkinson’s disease. The addition of Aβ and αSN to neurons triggered synapse damage at concentrations that did not affect neuronal survival [13], correlating with observations that synapse damage and clinical symptoms (due to the loss of neuron to neuron communication) can develop in these diseases before any gross neuronal loss is observed [2]. Such observations are consistent with the hypothesis that Aβ and αSN are responsible for the cognitive defects that occur during AD and Parkinson’s disease [2,10].

Drugs that maintain synapse function in the presence of Aβ and αSN may provide therapeutic benefit for patients when used as an adjunct to conventional Aβ lowering treatments. Here we show that some PDE inhibitors including pentoxifylline and caffeine protected neurons against Aβ and αSN-induced synapse damage. As some epidemiological studies concluded that regular consumption of coffee is associated with protection against AD, Parkinson’s disease and other dementias [21,22] and caffeine (1,3,7-trimethylxanthine) is the major active compound within coffee, it has been identified as a possible mediator of coffee’s neuroprotective effects. However, although caffeine protected neurons against Aβ and αSN-induced synapse damage, the concentrations required were higher than those normally found within the blood. The PDEs encompass a large family of proteins, each with specific cellular locations and substrate specificities. Currently, PDE4 is the subject of scrutiny for its role in neurodegenerative diseases as the selective PDE4 inhibitor rolipram improved cognitive functions in AD models [23,24,25]. Here we show that rolipram also protected neurons against Aβ and αSN-induced synapse damage. Sildenafil, a PDE5 inhibitor, also improved synaptic function in a mouse model of AD [26] suggesting the involvement of multiple PDEs in synapse function. Pentoxifylline did not protect neurons against a PLA_2_ derived from snake venom, which has a direct effect upon the pre-synaptic membrane [27] indicating that pentoxifylline acts of specific pathways.

Possible mechanisms of PDE inhibitor-mediated protection were examined. Aβ accumulates within synapses [28,29] and the high potency of Aβ oligomers suggests that their effects are mediated by specific receptors. However, the PDE inhibitors did not alter the concentrations of Aβ_42_ within synapses indicating that Aβ_42_ does not have a direct effect on synapses, rather that its actions are dependent upon specific pathways.

Our observations that Aβ and αSN increased the concentrations of cAMP in synaptosomes and reports that pre-synaptic cAMP and cAMP-dependent protein kinases affected neurotransmission [30,31] suggest that cAMP has a regulatory role at the synapse. As the PDEs degrade cAMP it was not surprising to observe that pentoxifylline and rolipram accentuated the Aβ and αSN-induced increase in cAMP in synaptosomes. Collectively these observations suggest that high concentrations of cAMP protect synapses, a hypothesis strengthened by our observation that cAMP analogues protected neurons against Aβ and αSN-induced synapse damage. The next step was to determine how cAMP might protect synapses. Both PDE inhibitors and cAMP analogues reduced the Aβ and αSN-induced activation of cPLA_2_ a key enzyme in synapse damage [14,19,32], in synaptosomes; an observation that is consistent with reports that cAMP inhibits PLA_2_ in other cell types [33].

We sought to determine the molecular mechanism underlying the Aβ and αSN-induced increase in cAMP. Selective cPLA_2_ inhibitors reduced the Aβ-induced increase in cAMP suggesting an inhibitory feedback effect whereby cAMP inhibits enzymes responsible for its own production. The activation of cPLA_2_ leads to the formation of prostaglandins and leucotrienes [34]. The Aβ-induced increase in cAMP was also reduced by pre-treating synaptosomes with the cyclo-oxygenase inhibitors aspirin and ibuprofen which prevent the production of prostaglandins. More specifically, PGE_2_, but not other PGs, increased synaptic cAMP concentrations and both Aβ and αSN-induced increases in synaptic cAMP were reduced by pre-treatment with a selective prostanoid E receptor antagonist indicating that PGE_2_ is the key prostaglandin regulating Aβ and αSN-induced synaptic cAMP production.

PGE_2_ has a complicated role at the synapse; although it regulates synaptic plasticity [35], higher concentrations trigger synapse damage [14]. We hypothesize that PGE_2_ acts as a feedback inhibitor of cPLA_2_ as illustrated in Figure 9. Thus, activation of cPLA_2_ leads to the production of PGE_2_ which increases the cAMP concentrations that have an inhibitory effect upon cPLA_2_ and hence its own production. This hypothesis was supported by our observations that PGE_2_ increased cAMP and significantly reduced the Aβ and αSN-induced activation of cPLA_2_. We propose that the inhibition of PDEs enhances the PGE_2_-induced rise in cAMP at the synapse, thus increasing the inhibition of cPLA_2_ and protecting the synapse.

## 5. Conclusions

In summary, we showed that some PDE inhibitors protect neurons against the synapse damage induced by Aβ or αSN. Their protective effect was associated with increased concentrations of cAMP in synapses and suppression of cPLA_2_, effects that were mimicked by cAMP analogues. We also demonstrate the presence of an inhibitory feedback system in which cAMP suppresses the activation of cPLA_2_ and the production of PGE_2_ and hence its own production.

## References

[1] Hardy J., Selkoe D.J. (2002). The amyloid hypothesis of Alzheimer’s disease: Progress and problems on the road to therapeutics. Science.

[2] Selkoe D.J. (2002). Alzheimer’s Disease Is a Synaptic Failure. Science.

[3] Hamos J.E., DeGennaro L.J., Drachman D.A. (1989). Synaptic loss in Alzheimer’s disease and other dementias. Neurology.

[4] Sze C.I., Troncoso J.C., Kawas C., Mouton P., Price D.L., Martin L.J. (1997). Loss of the presynaptic vesicle protein synaptophysin in hippocampus correlates with cognitive decline in Alzheimer disease. J. Neuropathol. Exp. Neurol..

[5] Haass C., Selkoe D.J. (2007). Soluble protein oligomers in neurodegeneration: Lessons from the Alzheimer’s amyloid β-peptide. Nat. Rev. Mol. Cell Biol..

[6] Lambert M.P., Barlow A.K., Chromy B.A., Edwards C., Freed R., Liosatos M., Morgan T.E., Rozovsky I., Trommer B., Viola K.L. (1998). Diffusible, nonfibrillar ligands derived from Aβ_1–42_ are potent central nervous system neurotoxins. Proc. Natl. Acad. Sci. USA.

[7] Klyubin I., Betts V., Welzel A.T., Blennow K., Zetterberg H., Wallin A., Lemere C.A., Cullen W.K., Peng Y., Wisniewski T. (2008). Amyloid-β Protein Dimer-Containing Human CSF Disrupts Synaptic Plasticity: Prevention by Systemic Passive Immunization. J. Neurosci..

[8] Lipton A.M., Cullum C.M., Satumtira S., Sontag E., Hynan L.S., White C.L., Bigio E.H. (2001). Contribution of asymmetric synapse loss to lateralizing clinical deficits in frontotemporal dementias. Arch. Neurol..

[9] Walsh D.M., Klyubin I., Fadeeva J.V., Cullen W.K., Anwyl R., Wolfe M.S., Rowan M.J., Selkoe D.J. (2002). Naturally secreted oligomers of amyloid β protein potently inhibit hippocampal long-term potentiation *in vivo*. Nature.

[10] Galvin J.E., Uryu K., Lee V.M., Trojanowski J.Q. (1999). Axon pathology in Parkinson’s disease and Lewy body dementia hippocampus contains alpha-, beta-, and gamma-synuclein. Proc. Natl. Acad. Sci. USA.

[11] Kramer M.L., Schulz-Schaeffer W.J. (2007). Presynaptic α-Synuclein Aggregates, Not Lewy Bodies, Cause Neurodegeneration in Dementia with Lewy Bodies. J. Neurosci..

[12] Lee V.M.Y., Trojanowski J.Q. (2006). Mechanisms of Parkinson’s Disease Linked to Pathological α-Synuclein: New Targets for Drug Discovery. Neuron.

[13] Bate C., Gentleman S., Williams A. (2010). α-synuclein induced synapse damage is enhanced by amyloid-β_1–42_. Mol. Neurodegener..

[14] Bate C., Tayebi M., Williams A. (2010). Phospholipase A_2_ inhibitors protect against prion and Aβ mediated synapse degeneration. Mol. Neurodegener..

[15] Shankar G.M., Li S., Mehta T.H., Garcia-Munoz A., Shepardson N.E., Smith I., Brett F.M., Farrell M.A., Rowan M.J., Lemere C.A. (2008). Amyloid-β protein dimers isolated directly from Alzheimer’s brains impair synaptic plasticity and memory. Nat. Med..

[16] Zhu J., Mix E., Winblad B. (2001). The antidepressant and antiinflammatory effects of rolipram in the central nervous system. CNS Drug Rev..

[17] Shelat P.B., Chalimoniuk M., Wang J.H., Strosznajder J.B., Lee J.C., Sun A.Y., Simonyi A., Sun G.Y. (2008). Amyloid beta peptide and NMDA induce ROS from NADPH oxidase and AA release from cytosolic phospholipase A_2_ in cortical neurons. J. Neurochem..

[18] Lehtonen J.Y., Holopainen J.M., Kinnunen P.K. (1996). Activation of phospholipase A_2_ by amyloid β-peptides *in vitro*. Biochemistry.

[19] Sanchez-Mejia R.O., Newman J.W., Toh S., Yu G.-Q., Zhou Y., Halabisky B., Cisse M., Scearce-Levie K., Cheng I.H., Gan L. (2008). Phospholipase A_2_ reduction ameliorates cognitive deficits in a mouse model of Alzheimer’s disease. Nat. Neurosci..

[20] Koch H., Huh S.E., Elsen F.P., Carroll M.S., Hodge R.D., Bedogni F., Turner M.S., Hevner R.F., Ramirez J.M. (2010). Prostaglandin E2-induced synaptic plasticity in neocortical networks of organotypic slice cultures. J. Neurosci..

[21] Rosso A., Mossey J., Lippa C.F. (2008). Caffeine: Neuroprotective functions in cognition and Alzheimer’s disease. Am. J. Alzheimers Dis. Other Dement..

[22] Ross G.W., Abbott R.D., Petrovitch H., Morens D.M., Grandinetti A., Tung K.H., Tanner C.M., Masaki K.H., Blanchette P.L., Curb J.D. (2000). Association of coffee and caffeine intake with the risk of Parkinson disease. JAMA.

[23] Gong B., Vitolo O.V., Trinchese F., Liu S., Shelanski M., Arancio O. (2004). Persistent improvement in synaptic and cognitive functions in an Alzheimer mouse model after rolipram treatment. J. Clin. Investig..

[24] Smith D.L., Pozueta J., Gong B., Arancio O., Shelanski M. (2009). Reversal of long-term dendritic spine alterations in Alzheimer disease models. Proc. Natl. Acad. Sci. USA.

[25] Sierksma A.S.R., van den Hove D.L.A., Pfau F., Philippens M., Bruno O., Fedele E., Ricciarelli R., Steinbusch H.W.M., Vanmierlo T., Prickaerts J. (2014). Improvement of spatial memory function in APPswe/PS1dE9 mice after chronic inhibition of phosphodiesterase type 4D. Neuropharmacology.

[26] Puzzo D., Staniszewski A., Deng S.X., Privitera L., Leznik E., Liu S., Zhang H., Feng Y., Palmeri A., Landry D.W. (2009). Phosphodiesterase 5 inhibition improves synaptic function, memory, and amyloid-beta load in an Alzheimer’s disease mouse model. J. Neurosci..

[27] Rigoni M., Caccin P., Gschmeissner S., Koster G., Postle A.D., Rossetto O., Schiavo G., Montecucco C., Montecucco C., Rossetto O. (2005). Equivalent effects of snake PLA_2_ neurotoxins and lysophospholipid-fatty acid mixtures. How do presynaptic PLA_2_ neurotoxins block nerve terminals?. Science.

[28] Gong Y., Chang L., Viola K.L., Lacor P.N., Lambert M.P., Finch C.E., Krafft G.A., Klein W.L. (2003). Alzheimer’s disease-affected brain: Presence of oligomeric Aβ ligands (ADDLs) suggests a molecular basis for reversible memory loss. Proc. Natl. Acad. Sci. USA.

[29] Lacor P.N., Buniel M.C., Chang L., Fernandez S.J., Gong Y., Viola K.L., Lambert M.P., Velasco P.T., Bigio E.H., Finch C.E. (2004). Synaptic Targeting by Alzheimer’s-Related Amyloid β Oligomers. J. Neurosci..

[30] Menegon A., Bonanomi D., Albertinazzi C., Lotti F., Ferrari G., Kao H.-T., Benfenati F., Baldelli P., Valtorta F. (2006). Protein Kinase A-Mediated Synapsin I Phosphorylation Is a Central Modulator of Ca2+-Dependent Synaptic Activity. J. Neurosci..

[31] Munno D.W., Prince D.J., Syed N.I. (2003). Synapse Number and Synaptic Efficacy Are Regulated by Presynaptic cAMP and Protein Kinase A. J. Neurosci..

[32] Desbene C., Malaplate-Armand C., Youssef I., Garcia P., Stenger C., Sauvee M., Fischer N., Rimet D., Koziel V., Escanye M.C. (2012). Critical role of cPLA(2) in Abeta oligomer-induced neurodegeneration and memory deficit. Neurobiol. Aging.

[33] Murthy K.S., Makhlouf G.M. (1998). Differential Regulation of Phospholipase A_2_(PLA_2_)-dependent Ca2+ Signaling in Smooth Muscle by cAMP- and cGMP-dependent Protein Kinases. J. Biol. Chem..

[34] Sun G.Y., Xu J., Jensen M.D., Simonyi A. (2004). Phospholipase A_2_ in the central nervous system: Implications for neurodegenerative diseases. J. Lipid Res..

[35] Chen C., Bazan N.G. (2005). Endogenous PGE_2_ Regulates Membrane Excitability and Synaptic Transmission in Hippocampal CA1 Pyramidal Neurons. J. Neurphysiol..

